# Supplementary material for: Harmonizing material quantity and terahertz wave interference shielding efficiency with metallic borophene nanosheets
Source: Nat Commun. 2025 Jul 1;16:5739. doi: 10.1038/s41467-025-60892-1 (PMC12218330; doi:10.1038/s41467-025-60892-1)
Supplement: Supplementary file 1 — Supplementary Information [file 41467_2025_60892_MOESM1_ESM.pdf]

Supplementary Information

Harmonizing Material Quantity and Terahertz Wave Interference Shielding Efficiency with Metallic Borophene Nanosheets

Haojian Lin[1], Ximiao Wang[1], Hongjia Zhu[1], Zhaolong Cao, Jiahao Wu, Runze Zhan, Ningsheng Xu, Shaozhi Deng*, Huanjun Chen*, and Fei Liu*

State Key Laboratory of Optoelectronic Materials and Technologies, Guangdong Province Key Laboratory of Display Material and Technology, and School of Electronics and Information Technology, Sun Yat-sen University, Guangzhou 510275, China

*Corresponding authors' e-mail: liufei@mail.sysu.edu.cn; chenhj8@mail.sysu.edu.cn; stsdsz@mail.sysu.edu.cn.

Supplementary Table 1 | Comparison of EMI SE performances of various THz shielding materials.

Materials	Weight ratios (wt.%)	Thickness (mm)	Frequency (THz)	EMI SE (dB)	EMI EES_t ($dB\cdot cm^2\cdot g^{-1}$)	Ref.
MXene film	–	0.0005	0.25–2.00	30	–	1
MWCNTs film	–	~0.02	0.40–2.20	30	–	2
α-Br/PDMS	~33.33	5	0.10–2.70	~42	0.125	3
MXene/PAA/ACC	8.5	0.13	0.20–2.00	45.3	–	4
α-Br tablet	–	1	0.10–2.70	50	–	3
Gr/Cu	–	0.00016	0.10–1.00	60.95	–	5
MXene film	–	0.025	0.30–0.70	70	–	6
rGO paper	–	~0.37	0.10–1.00	72.1	–	7
OLC/PMMA	2	0.12	0.1–3	4	3.4	8
SWCNT film on PET film	–	5×10^{-8}	0.1–1.2	4	3.6	9
SWCNTs/PVA	1.6	0.3	0.3–2.1	29	15.7	10
MWCNTs/PMMA	~2	~0.48	0.10–4.00	20	16.9	11
CNF/PTFE/PVDF/PMMA	~10	~0.05	0.57–0.63	32	27.1	12
Gr/PDMS	10	~2	0.10–0.80	~32	30	13
CNW/PMC	50	~0.03	0.57–0.63	~40	36.4	14
PAN/PU	10	0.15	0.2–1.2	42	38.2	15
Graphite/PMMA	35.7	0.30	0.20–0.90	~50	42.4	16
Kapton-derived carbon	–	0.125	0.22–0.5	~70	54.5	17
Zn^{2+}/MXene/GO foams	–	0.085	0.20–2.00	~51	451	18
GO/Fe_3O_4	500	10	0.10–2.50	–	16000	19
Gr-1500/MWCNT	–	3	0.10–1.60	40	21000	20
MXene/GO foams	20	4	0.20–2.00	–	46000	21
Gr foams-1500	–	3	0.10–1.60	50	110000	20
Gr/PMMA	–	0.034	0.1–1.00	60	300000	22
3D Gr	–	–	0.1–3	35	30000	23
Gr/TiCN@PI/MXene	–	0.016	0.5–1.5	45	900	24
Fe_3O_4/Gr	27.3	10	0.1–2.5	42	16000	19
Fe_3O_4/Polymer/CNFs	40	0.54	0.1–1.2	~60	–	25
MXene/CNT Janus	–	0.00005	0.3–1.6	60	230756	26
MXene/PGPDMS	83.3	0.12	0.5–3	57.5	24000	27
Aramid/MXene Janus	80	0.9	0.2–2.4	60.49	–	28
Polymer/Cellulose	50	6.9	0.2–1.2	~58	66493.99	29
3D MXene/PI	80	0.11	0.6–1.1	70.4	–	30
CNFs/Polyme	50	~0.6	0.1–1.2	43.9	–	31
Gr/Fe_3GeTe_2/$FeTe_2$/Fe_3Ge	–	2.5	0.5–1.6	76	–	32
Gr-220/700	5	2	0.2–1.2	56.6	–	33
PA/TS@IL-Ag-rGO	3	–	0.2–2	36	–	34
PUS-Ni/MXene	~53	8	0.15–2.15	69.8	65.8	35
MXene/PPy	91	0.04	0.2–1.6	71.4	36983	36
MXene/Gr	75	0.048	0.1–1	60	–	37
CNF/SBC	20	3	0.4–2	70	–	38
OCF/GO	100	4.34	0.3–1.5	34	–	39
MXene-PMMA/rGO-PVP	16.7	0.148	0.37–2	57.7	–	40
MS-Ni/CNT	51.6	8	0.1–2.2	79.1	66.3	41
**β_{12}-Br/PDMS**	**0.13**<br>**0.50**	**2**<br>**4**	**0.10–2.00**	**68**<br>**83**	**480000**<br>**250000**	**This work**

Note: MXene ($Ti_3C_2T_x$), GO (graphene oxide), PAA (poly (acrylic acid)), ACC (amorphous calcium carbonate), CNF (carbon nano fiber), PTFE (polytetrafluoroethylene), PVDF (poly (vinylidene fluoride)), PMMA (poly (methyl methacrylate)), CNW (carbon nano whiskers), PMC (fluor acrylic copolymer), MWCNTs (multiwalled carbon nanotubes), PVA (polyvinyl alcohol), PDMS (polydimethylsiloxane), OLC (onion-like carbon), PAN (polyaniline), PU (polyurethane), SBC (sustainable biocarbon), OCF (oxidized carbon fiber), MS (melamine sponge), PVP (polyvinylpyrrolidone), Gr (graphene), PPy (polypyrrole), PUS (polyurethane sponge), PA/TS@IL (polyamide elastomer/towel-gourd sponge@ionic liquids), PI (polyimide), PGPDMS (poly-(3-glycidox-ypropyldimethoxymethylsilan)), PET (polyethylene terephthalate).

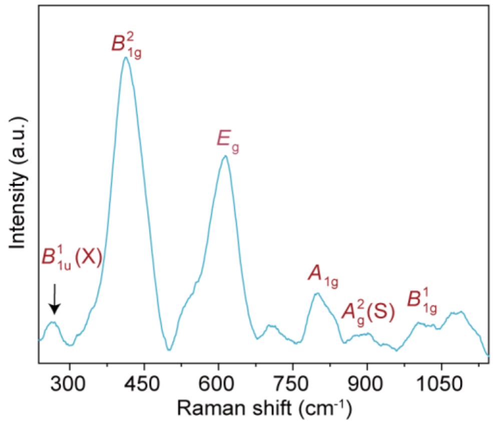

Supplementary Fig. 1 | A typical Raman spectrum of *β*12-Br nanosheets.

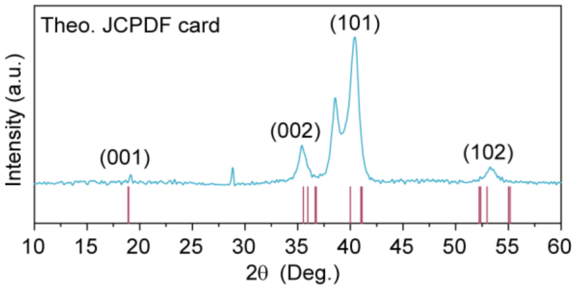

Supplementary Fig. 2 | XRD pattern of *β*12-Br nanosheets. The XRD pattern demonstrates a nice agreement between the characteristic peaks of the 2D nanosheets and the theoretical XRD peaks obtained from the DFT calculations. Furthermore, the narrower full width at half maximum (FWHM) and sharper diffraction peaks observed in the *β*12-Br nanosheets reveal their high-quality crystallinity.

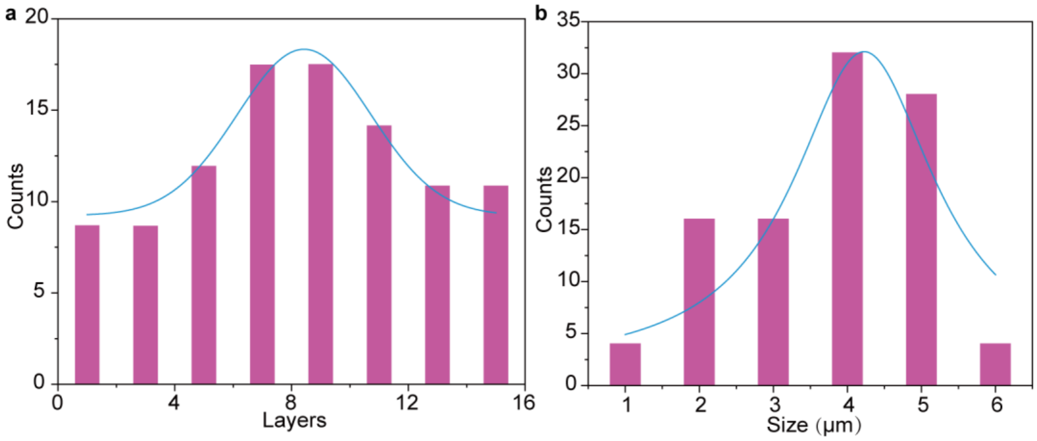

Supplementary Fig. 3 | The layer-number (a) and lateral size (c) distribution diagram of the as-grown *β*12-Br nanosheets.

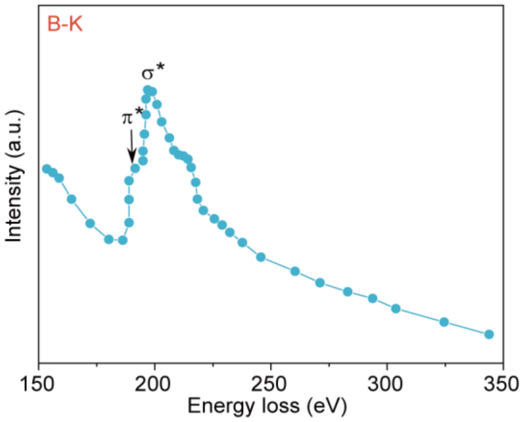

Supplementary Fig. 4 | Representative EELS spectrum of a *β*12-Br nanosheet. There is an obvious characteristic peak at 197.1 eV in the EELS spectrum of the as-grown nanosheet, which should originate from the boron K-shell[42]. Also, the atomic ratio of element B in the nanosheet is over 98 at.%. It is therefore suggested that the nanosheet can be indexed as 2D *β*12-Br with a planar sp^2 bonding configuration resemble to graphene[43].

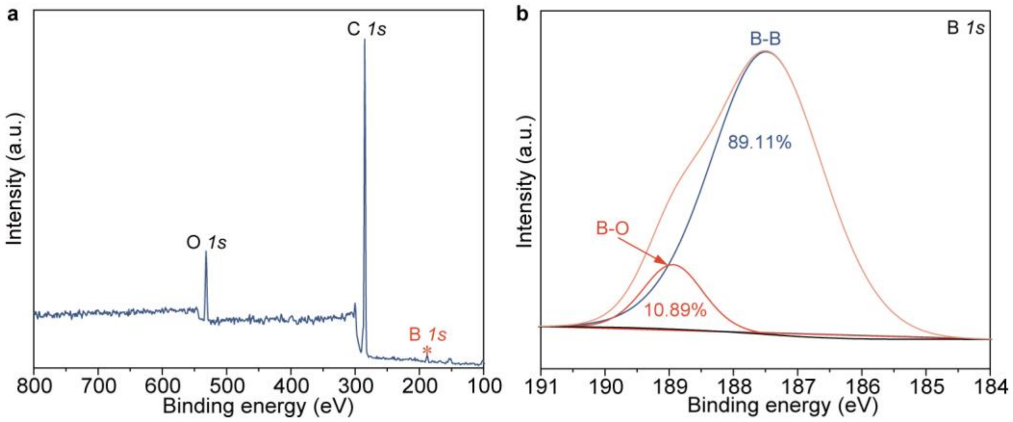

Supplementary Fig. 5 | XPS spectra of *β*12-Br nanosheets. a Full-scale survey spectrum. **b** High-magnification XPS spectrum of B 1s. Three characteristic peaks of C 1s, O 1s, and B 1s are clearly found in the XPS spectrum. From the high-resolution XPS spectra (**b**), B 1s peak can be seen to be consisted of both B–O and B–B signal peaks and the molar ratio of B–B is over 90%.

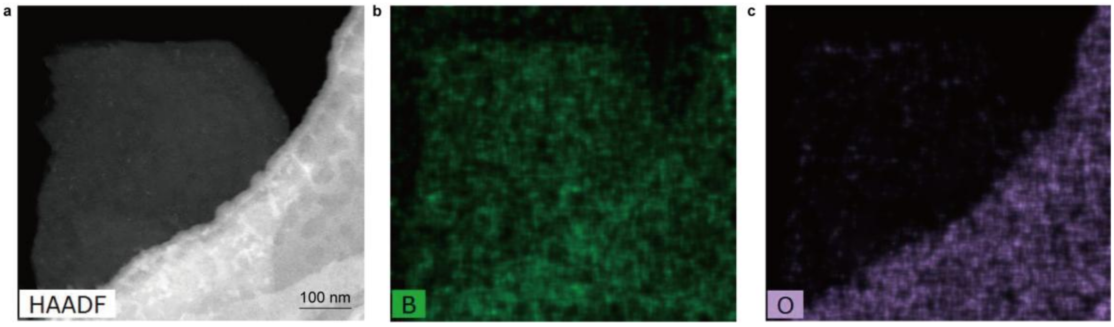

Supplementary Fig. 6 | a Typical HAADF-STEM image of a β_{12}-Br nanosheet. **b, c** The corresponding EDX mapping images of boron and oxygen elements, respectively. It is clearly seen that element B uniformly distributes over the nanosheet whereas element O only exists at the margin of the nanosheet. This suggests a slight oxidation of boron atoms occurrence at the edge of borophene nanosheets.

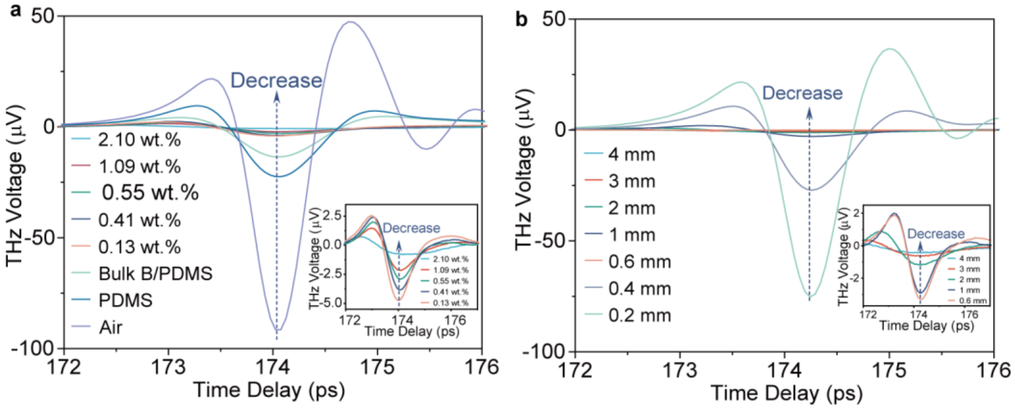

Supplementary Fig. 7 | THz-TDS spectra of β_{12}-Br/PDMS composite films with different weight ratios of boron fillers (a) and various film thicknesses (b). It can be observed that a 2-mm-thickness boron/PDMS composite film with 1.09 wt.% bulk boron powders only reduces the absolute THz voltage from 22.9 to 13.8 mV in comparison with a pure PDMS film with the same thickness. When adding 0.13 wt.% β_{12}-Br nanosheets into PDMS gel, the absolute THz voltage of the 2-mm-thickness composite film significantly decreases from 22.9 to 4.7 mV, unveiling that the β_{12}-Br nanosheets exhibit superior performance in THz shielding than bare bulk boron powders. Furthermore, as found in Fig. S7a, the absolute THz voltage of β_{12}-Br/PDMS composite film will decrease with increasing the weight ratios of β_{12}-Br nanosheets when the thickness of composite film is kept at 2 mm, suggesting that higher concentrations of β_{12}-Br nanosheets will provide more effective shielding against THz waves. Additionally, the effect of the composite film's thickness on its THz shielding

behaviors should not be ignored. TDS spectra for β_{12}-Br/PDMS composite films with different thicknesses are shown in Fig. S7b, where the weight ratios of β_{12}-Br nanosheets remains at 0.50 wt.%. When reducing the thickness of the composite film from 4 to 0.20 mm, the absolute THz voltage is seen remarkably increase from 0.70 to 118 mV, indicating that the composite film's thickness is a crucial factor for its THz shielding performance.

Supplementary Note 1 | Discussion on the saturation of the EMI SE with increasing of the borophene weight fraction

As shown in Fig. 4b in the main text, by progressively increasing the borophene content from 0.13 to 2.1 wt.%, the EMI SE initially experiences rapid augmentation, eventually reaching a plateau for weight fraction over 0.5 wt.%. Such saturation effect can be attributed to the competition between absorption and scattering of incident THz wave by the β_{12}-Br nanosheets at different borophene content. According to our previous study[44] and that from other research group[45], THz wave absorption by the β_{12}-Br nanosheets will monotonically increase with the borophene content. When the borophene content arrives at a critical value, the maximum THz absorbance of the β_{12}-Br nanosheets will be achieved. But if the borophene content is continuously increased to exceed the critical value, the scattering coefficient will sharply increase, originating from the strong scattering loss of the incident THz waves by the nanosheet interface instead of being fully absorbed by β_{12}-Br nanosheets. This will lead to suppression of THz wave absorption and therefore saturation of the EMI SE.

This behavior can be quantitatively described using **coupled mode theory**[44, 45], which describes the absorption, reflection, and transmission spectra of the β_{12}-Br nanosheets as a function of their contents within the PDMS matrix. Fig. S8 shows a schematic diagram of the borophene nanosheets dispersed in a PDMS matrix. To simplify the discussion, the nanosheets are approximated as rectangular array. Here, $\varepsilon_1 = \varepsilon_2$ represent the dielectric functions of the PDMS separated by the interface.

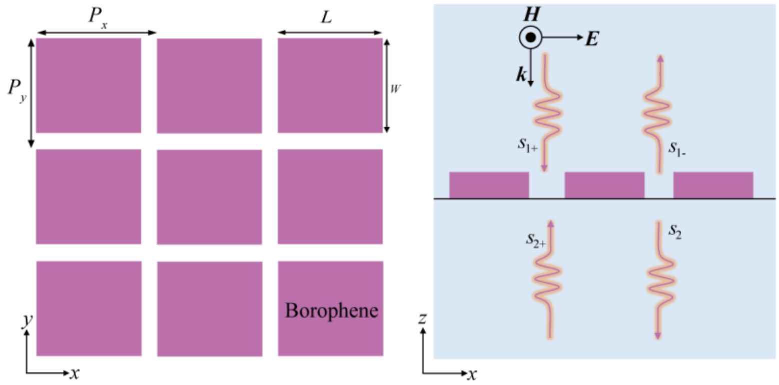

Supplementary Fig. 8 | Schematic diagram showing the calculation of the absorption, reflection, and transmission spectra of the β12-Br nanosheets embedded into the PDMS matrix using the coupled mode theory.

The perpendicularly incident THz waves can be expressed as $|s_+\rangle = [s_{1+} s_{2+}]^T$, while the outgoing THz waves can be written as $|s_-\rangle = [s_{1-} s_{2-}]^T$, where $s_{1+}(s_{1-})$ and $s_{2+}(s_{2-})$ are the complex amplitudes of the incident (outgoing) wave in the PDMS matrix. The time-varying mode amplitude, a, of the β12-Br sheets can be written as,

$$\begin{cases} \dfrac{da}{dt} = j(\omega_0 + j\Gamma_{tot}/2)a + (\langle d|^*)|s_+\rangle \\ |s_-\rangle = C|s_+\rangle + a|d\rangle \end{cases} \tag{S1}$$

where the total decay rate (Γ_{tot}) is the sum of THz radiative decay rate (Γ_{rad}) and the THz absorbance (Γ_{abs}), and $|d\rangle = [d_1 d_2]^T$ is the coupling vector under the condition of $\langle d|d\rangle = \Gamma_{rad}$. Parameter C is the background scattering of PDMS matrix without the β12-Br nanosheets, which can be depicted as,

$$C = \begin{bmatrix} r_0 & jt_0 \\ jt_0 & r_0 \end{bmatrix} \tag{S2}$$

where $r_0 = \dfrac{\sqrt{\varepsilon_2} - \sqrt{\varepsilon_1}}{\sqrt{\varepsilon_1} + \sqrt{\varepsilon_2}}$ and $t_0 = \dfrac{2\sqrt{\sqrt{\varepsilon_1 \varepsilon_2}}}{\sqrt{\varepsilon_1} + \sqrt{\varepsilon_2}}$ are the background reflection and transmission coefficients, respectively. According to the time-reversal symmetry and

conservation of energy, $CC^{+} = \boldsymbol{I}$ and $C|d\rangle^{*} = -|d\rangle$, parameters d_1 and d_2 can be expressed as,

$$\begin{cases} d_1 = -\sqrt{\dfrac{\Gamma_{rad}(1-r_0)}{2}} \\ d_2 = j\sqrt{\dfrac{\Gamma_{rad}(1+r_0)}{2}} \end{cases} \tag{S3}$$

By solving the above differential equation, the time-varying mode amplitude (a) can be written as,

$$a = \frac{(\langle d|^{*})s_{+}\rangle}{j(\omega - \omega_0 - j\Gamma_{tot}/2)} \tag{S4}$$

When the THz wave incidents vertically onto the interface between PDMS and β_{12}-Br nanosheets from the upper space of the PDMS matrix, the reflection (r) and transmission (t) coefficients of β_{12}-Br nanosheets can be obtained as,

$$\begin{cases} r = \dfrac{s_{1-}}{s_{1+}} = r_0 + \dfrac{\Gamma_{rad}(1-r_0)/2}{j(\omega-\omega_0)+\Gamma_{tot}/2} \\ t = \dfrac{s_{2-}}{s_{1+}} = jt_0 - \dfrac{j\Gamma_{rad}t_0/2}{j(\omega-\omega_0)+\Gamma_{tot}/2} \end{cases} \tag{S5}$$

Therefore, the total absorption, A, of the β_{12}-Br nanosheets embedded into the PDMS matrix can be deduced as[44],

$$A = 1 - |r|^2 - |t|^2 = \frac{|a|^2 \Gamma_{abs}}{|s_{1+}|^2} = \frac{\Gamma_{abs}\Gamma_{rad}(1-r_0)/2}{(\omega-\omega_0)^2 + (\Gamma_{tot}/2)^2} \tag{S6}$$

According to Eq. S6, the absorption, A of the β_{12}-Br nanosheets is a function of the radiative decay rate and absorbance decay rate. For nanosheets with sizes much smaller than the wavelength of the THz wave, which is the case in our current study, the radiative decay rate can be approximated as[44],

$$\Gamma_{rad} = \frac{\zeta_1^2 E_F e^2}{2\pi\varepsilon_0 c\bar{n}\hbar^2}\frac{D^2}{S} \tag{S7}$$

where e is the charge of an electron, $\hbar$ is the reduced Planck constant, $\bar{n}$ is the average refractive index of the surrounding medium, ζ_1 is approximated as a constant for the longitudinal plasmonic dipole mode, E_F is Fermi energy, c is the speed of light, ε_0 is the vacuum permittivity, $D = \sqrt{LW}$ is the characteristic size of the borophene nanosheet, S is the lattice area with $S = P_xP_y$. Therefore, Γ_{rad} is determined by the duty ratio, D^2/S, of β_{12}-Br nanosheet arrays, which is proportional to the borophene weight fraction. The maximum absorption is achieved under the condition $\partial A/\partial\Gamma_{rad} = 0$, yielding the well-known critical coupling condition $\Gamma_{rad} = \Gamma_{abs}$. Consequently, when measuring the absorption of the β_{12}-Br/PDMS composite, it initially increases and reaches a maximum value once the borophene content corresponding to the critical coupling condition is achieved. Beyond this point, the absorption strength, which is proportional to the EMI SE, begins to decrease. This qualitatively explains the experimental results shown in Fig. 4b of the original manuscript, where the THz EMI SE of the β_{12}-Br/PDMS film initially increases with borophene content and then gradually saturates.

Another reason for the saturation behavior can be attributed to the detection limit of the terahertz time-domain spectroscopy (THz-TDS) system used in our study. According to the shielding efficiency equation, EMI SE (dB) = $20\log_{10}\left(\frac{I_{in}}{I_{out}}\right)$, where I_{in} an I_{out} represent the intensities of the incident and transmitted THz waves, respectively. When the borophene content reaches approximately 0.5 wt.%, the THz EMI SE achieves a value of up to 80 dB. This corresponds to 99.99% of the incident wave being absorbed, with only 0.01% of the wave transmitting through the composite film. The detection limit of the THz-TDS spectrometer used in our measurements (Model: TDS, BATOP, TDS 1008) is 85 dB. This means that when the transmitted wave intensity falls below 5×10^{-3}% of the incident wave, the spectrometer can no longer reliably distinguish intensity differences associated with varying transmittance levels. As a result, the THz EMI SE of the β_{12}-Br/PDMS film appears to saturate in our experiments once the borophene content exceeds 0.5 wt.%, remaining nearly constant

with further increases in borophene content.

Supplementary Note 2 | Numerical simulations on the THz responses of β_{12}-Br nanosheet and its arrays

We employed the finite-difference time-domain (FDTD, Lumerical) method to calculate the near-field distribution, charge distribution, and Joule heat loss within an individual borophene circular nanosheet, as shown in Fig. 4f–h in the main text. The THz absorption cross-sections of various individual nanosheets and absorption spectra of the corresponding nanosheet arrays were calculated using the finite element method (COMSOL, MULTIPHYSICS). To simplify the calculation process and conserve computational resources, a perfect 2D disk model without a thickness parameter is used in our calculations.

One pivotal parameter is the complex conductivity of borophene. However, to our best knowledge, there is no literature reports on the conductivity of borophene in the THz domain. Considering that our synthesized β_{12}-Br nanosheets exhibit a very high conductance, we employed Drude model, which describes the electromagnetic responses of free electrons, to calculate the conductivity of borophene, which is assumed to be isotropic and written as[46],

$$\sigma = \frac{iD}{\pi\left(\omega + \frac{i}{\tau}\right)} \tag{S8}$$

$$D = \frac{\pi e^2 n}{m^*} \tag{S9}$$

where n, ω, τ, D, and m^* stand for electron charge, electron density, frequency of THz wave, carrier lifetime, Drude weight, and effective electron mass.

As an initial approximation, m^* is adopted as $m^* = 1.4\times m_0 = 1.274\times10^{-30}$ kg. The carrier density n and relaxation time τ are adopted from the theoretical studies, with values of 3.4×10^{19} cm^{-2} and 65 fs[46, 47], respectively. The resulting conductivity is depicted in Fig. S9a and S9b (black curves). The subsequent step involves comparing the simulation results with experimental data to assess their consistency. To this end, we calculate the THz absorption spectra of a disk array. Based on morphological characterization (Fig. 1c in the original manuscript), most borophene nanosheets exhibit

irregular shapes. Therefore, a square disk array is employed as a simplified model for the simulation, as illustrated in Fig. S9c. Specifically, the side length of each disk is set to 5 μm, a value determined by the measured average lateral size of the nanosheets, as illustrated in Fig. S3b. This choice ensures that the model accurately reflects the physical dimensions of the synthesized β_{12}-Br nanosheets. The separation between adjacent disks is set to 20 μm to avoid potential near-field electromagnetic coupling. The disks are arranged in a 2D square lattice. The surrounding refractive index is set as 1.53 to model the PDMS matrix. Using the initial conductivity, the calculated absorption spectrum of the square disk array reveals a resonance around 8.7 THz (Fig. S9d), which significantly deviates from the experimental measurements. The experimental data for the β_{12}-Br/PDMS composite film show strong THz absorption around 0.87 THz (Fig. 4a in the main text). This discrepancy suggests that the conductivity parameters need to be adjusted to achieve better agreement between the simulation and experimental results.

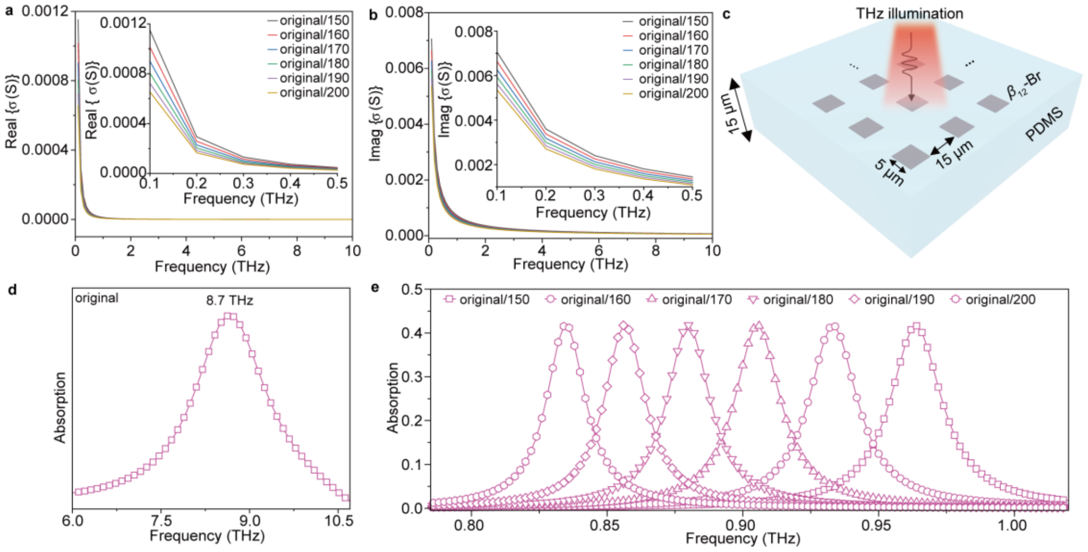

Supplementary Fig. 9 | a, **b** Real part (**a**) and imaginary part (**b**) of β_{12}-Br conductivity at different correction coefficient ξ, respectively. **c** Schematic showing the simulation model. **d** Simulated THz absorption spectrum of 2D β_{12}-Br nanosheet array, where τ is 65 fs, $n = 3.4 \times 10^{19}$ m^{-2}. **e** THz absorption spectra of 2D β_{12}-Br nanosheet arrays using the conductivities at different ξ. The arrays are all placed inside a PDMS environment.

Given that the β_{12}-Br nanosheets in our study were synthesized using a wet chemical method, unintentional doping and structural defects are likely to be introduced. These factors will result in carrier density and relaxation time values that differ from

those previously reported. Our goal is to redshift the resonance frequency of the nanosheet array to a lower range, which requires a reduction in electron density, as supported by plasmonic theory[48]. To achieve this, we introduce a correction factor ξ to adjust the electron density, expressed as $n = 3.4 \times 10^{19}/\xi$ cm^{-2}. Reducing n will consequently lower the electron scattering rate, leading to a corresponding adjustment in the relaxation time as $\tau = 65\xi$. This approach allows us to fine-tune the conductivities (Fig. S9a and S9b, color lines) to better align the simulated resonance frequency with the experimental observations. As shown in Fig. S9d and S9e, the resonance peak of the β_{12}-Br nanosheet arrays shifts from 8.7 THz to approximately 0.88 THz at $\xi = 180$, which is consistent with the experimental result (0.87 THz). Therefore, a ξ of 180 is employed in all of the simulations in our manuscript (Table S2).

Once the conductivity has been determined, we can proceed to calculate the THz wave responses of nanosheets with varying shapes and sizes. This includes evaluating the absorption cross-section, near-field distribution, charge distribution, and Joule loss within an individual nanosheet. Fig. S10 presents the calculated absorption cross-sections for nanosheets with different shapes (Fig. S10a) and sizes (Fig. S10b). The results demonstrate that the β_{12}-Br nanosheets exhibit strong resonances originated from the free electrons, whereby the resonance frequency is highly sensitive to its size and shape, allowing for tunability across a broad spectral range. In addition, for nanosheet arrays, the maximum absorption efficiency remains nearly constant at 43%, regardless of the geometrical parameters of the borophene nanosheets (Fig. S10c). Consequently, the broadband yet relatively stable THz EMI absorption shielding performance observed in our experimental measurements for the β_{12}-Br/PDMS composite film can be attributed to the presence of nanosheets with varying thicknesses and shapes within the composite film. This diversity in nanosheet morphology contributes to the consistent and wide-ranging absorption characteristics of the material.

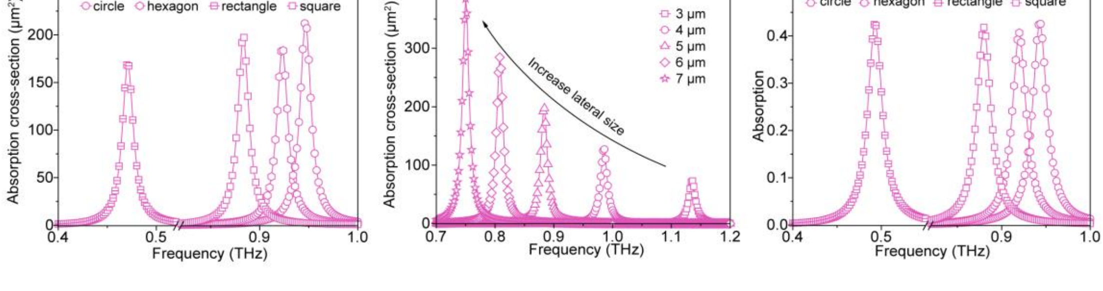

Supplementary Fig. 10 | a Calculated absorption cross-sections for nanosheets with circular, hexagon, rectangle, and square shapes. The areas of these nanosheets are set

as 25 μm^2. **b** Calculated absorption cross-sections for square nanosheets of different side lengths. **c** Calculated absorption spectra of nanosheet arrays with different shapes. The areas of the nanosheets are all set as 25 μm^2. The separations between adjacent nanosheets in these arrays are set as 20 μm.

The THz near-field characteristics of a specific nanosheet can also be readily calculated once its absorption cross-section spectrum is obtained. According to the experimental measurement shown in Fig. 4i in the main text, the diameter of the disk is set as 5.64 μm. The disk is placed onto 500-μm-thick highly doped *n*-Si substrate, exactly as that used in the experimental near-field measurement. The surrounding environment is set as vacuum, with a refractive index of 1.0. The refractive index ($\tilde{n}$) of *n*-doped silicon was written as[49],

$$\tilde{n}^2 = \varepsilon_{Si} - \frac{\omega_p^2}{\omega(\omega + i\Gamma)} \quad \text{(S10)}$$

$$\omega_p^2 = \frac{N_c e^2}{\varepsilon_0 m_{Si}^*} \quad \text{(S11)}$$

where ε_{Si}, Γ, and N_c equal 11.66, 6.141 THz, and 2 × 10^{16} cm^{-3}, respectively. Parameter ε_0 is the vacuum permittivity, m_{Si}^* is adopted as m_{Si}^* = 0.26 × m_0 = 2.368 × 10^{-31} kg. To calculate the near-field distribution and compare it with the experimental near-field measurement, the scanning tip of the scattering-type scanning near-field optical microscope was modeled as a point dipole source raster-scanned across the simulation domain. The dipole, oriented perpendicular to the sample surface, was positioned at a fixed height of 200 nm above the substrate. For each simulation, the perpendicular component of the electric field, $|E_z|$, was extracted in the frequency domain (0.5 to 2.0 THz) at a plane 100 nm above the surface and converted back to time-domain near-field optical maps via inverse Fourier transform. The $|E_z|$ was then integrated over the time domain to obtain the near-field distribution, as shown in Fig. 4h in the main text. For calculation of the charge density (ρ) and Joule loss (J) within the nanosheet, a broadband plane wave was employed to illuminate the nanosheet, whereby the electric field, $\boldsymbol{E}$, within the nanosheet was recorded Afterwards, ρ and J can be obtained according to,

$$\rho = \varepsilon_0 \nabla \cdot \vec{E} \tag{R12}$$

$$J = \frac{1}{2}\sigma\vec{E} \cdot \vec{E}^* \tag{R13}$$

To ensure consistency with the experimental conditions, we employed the same broadband source (0.5 to 2.0 THz) in our simulations to excite an individual borophene nanosheet and performed spectral integration of the ρ and J over the same spectral range.

To calculate the absorption spectra of β_{12}-Br nanosheet arrays with different nanosheet thicknesses to demonstrate the absorption spectrum changes against the nanosheet thickness. The three-dimensional (3D) dielectric function of the borophene nanosheet is described as[50],

$$\varepsilon_r = \varepsilon_\infty - \frac{e^2 n}{m^* \varepsilon_0 d\left(\omega^2 + \frac{1}{\tau^2}\right)} \tag{S14}$$

$$\varepsilon_i = \frac{e^2 n / \tau}{m^* \varepsilon_0 d\omega\left(\tau\omega^2 + \frac{1}{\tau^2}\right)} \tag{S15}$$

where ε_r and ε_i are respectively the real and imaginary parts of the complex dielectric function, $\varepsilon_\infty = 11$ is the relative permittivity, $\varepsilon_0 = 8.854\times10^{-12}$ F·m^{-1} is the vacuum permittivity, and d represents the thickness of β_{12}-Br nanosheet. Eqs. S14 and S15 give the thickness-dependent dielectric function of the β_{12}-Br nanosheet, whereby the THz absorption spectra of the nanosheet arrays with different borophene thicknesses can be readily calculated.

Supplementary Table 2 | Parameters used in the numerical simulations.

Parameters	Values
n	1.89×10^{17} m^{-2}
τ	11.7 ps
m^*	1.274×10^{-30} kg
Refractive index of PDMS	1.53
Refractive index of the surrounding vacuum	1.00
Permittivity of the highly doped n-Si substrate (ε_{Si})	11.66
Side length of square disk used in the absorption spectra simulations shown in Fig. S9d	5.0 μm
Side length of square disk used in the absorption spectra simulations shown in Fig. S9e	5.0 μm
Diameter of circular disk used in the near-field responses simulations shown in Fig. 4f, 4g, and 4h in the main text in the main text	5.64 μm
Diameter of circular disk used in the absorption cross-sections simulations shown in Fig. S10a	5.64 μm
Side length of square disk used in the absorption cross-sections simulations shown in Fig. S10a	5.0 μm
Side length of hexagon disk used in the absorption cross-sections simulations shown in Fig. S10a	3.10 μm
Side length of rectangle disk used in the absorption cross-sections simulations shown in Fig. S10a	10 μm × 2.5 μm
Side length of square disk used in the absorption cross-sections simulations shown in Fig. S10b	3 to 7 μm, with an increment of 1 μm
Diameter of circular disk used in the absorption spectra simulations shown in Fig. S10c	5.64 μm
Side length of square disk used in the absorption spectra simulations shown in Fig. S10c	5.0 μm
Side length of hexagon disk used in the absorption spectra simulations shown in Fig. S10c	3.10 μm
Side length of rectangle disk used in the absorption spectra simulations shown in Fig. S10c	10 μm × 2.5 μm
Separation between adjacent disks in the array	20 μm
Thicknesses of all of the disks used in simulations of Fig. S9, Fig. S10, and Fig. 4f, 4g, and 4h in the main text	0

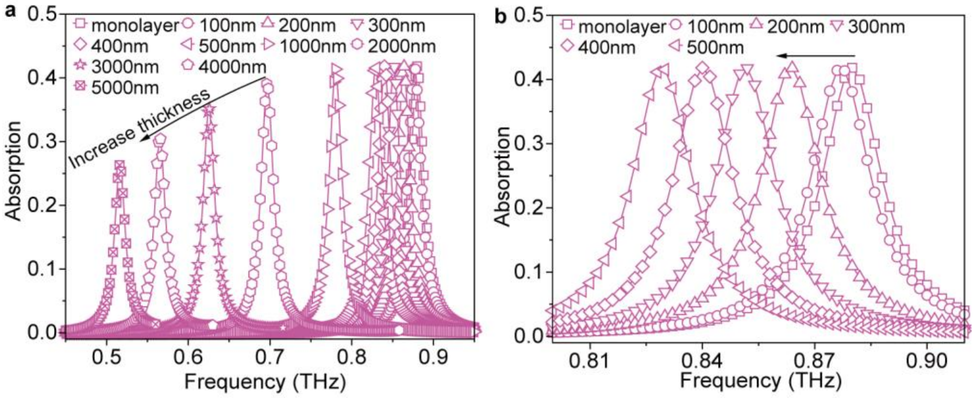

Supplementary Fig. 11 | a Simulated results of the absorption spectra of β_{12}-Br nanosheet arrays of different nanosheet thicknesses. **b** Enlarged spectra in the frequency range of 0.8 to 0.91 THz. The nanosheets are of square shape and arranged into square lattice, with side length of 5 μm. The separation between adjacent nanosheets are 20 μm. The arrays are placed inside PDMS matrix.

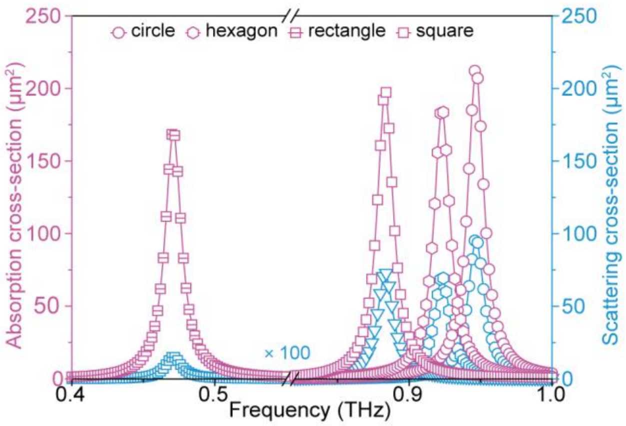

Supplementary Fig. 12 | Calculated absorption (purple) and scattering (blue) cross-sections for nanosheets with circular, hexagon, rectangle, and square shapes. The areas of these nanosheets are set as 25 μm^2. The scattering cross-sections are multiplied by 100 times for a better comparison with the absorption cross-sections, showing that the scattering of an individual nanosheet is much smaller than its absorption.

Supplementary Table 3 | Tensile properties of different 2D material-based composites for THz EMI shielding.

Materials	Tensile Stress (Pa)	Strain (%)	Ref.
MXene/PAA/ACC hydrogels	20k	1500	4
MXene/PVA hydrogels	100k	50	51
Gr/PMMA	~48M	~8	22
GO/PMMA	~26M	~2.2	52
**β_{12}-Br/PDMS**	**~32M**	**~158**	**This work**

Note: MXene ($Ti_3C_2T_x$), GO (graphene oxide), PAA (poly (acrylic acid)), ACC (amorphous calcium carbonate), Gr (graphene).

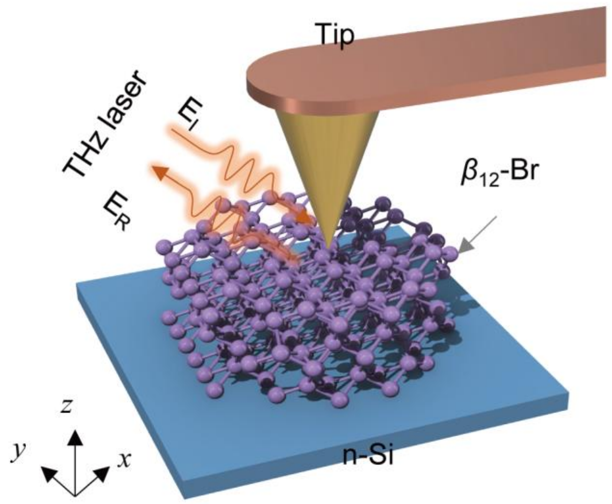

Supplementary Fig. 13 | Schematic showing operation principle of the near-field THz-TDS system.

References

1. Choi, G., et al. Enhanced terahertz shielding of MXenes with nano-metamaterials. *Adv. Opt. Mater.* **6**, 1701076 (2018).
2. Polley, D., Neeraj, K., Barman, A. & Mitra, R. K. Diameter-dependent shielding effectiveness and terahertz conductivity of multiwalled carbon nanotubes. *J. Opt. Soc. Am. B* **33**, 2430-2436 (2016).
3. Zhang, Z., et al. Few-layer borophene prepared by mechanical resonance and its application in terahertz shielding. *ACS Appl. Mater. Interfaces* **12**, 19746-19754 (2020).
4. Zhu, Y., et al. Multifunctional $Ti_3C_2T_x$ MXene composite hydrogels with strain sensitivity toward absorption-dominated electromagnetic-interference shielding. *ACS Nano* **15**, 1465-1474 (2021).
5. Hou, S., et al. Excellent terahertz shielding performance of ultrathin flexible Cu/graphene nanolayered composites with high stability. *J. Mater. Sci. Technol.* **52**, 136-144 (2020).
6. Zou, Q., et al. MXene-based ultra-thin film for terahertz radiation shielding. *Nanotechnology* **31**, 505710 (2020).
7. Dong, S., Shi, Q., Huang, W., Jiang, L. & Cai, Y. Flexible reduced graphene oxide paper with excellent electromagnetic interference shielding for terahertz wave. *J. Mater. Sci.: Mater. Electron.* **29**, 17245-17253 (2018).
8. Macutkevic, J., et al. Terahertz probing of onion-like carbon-PMMA composite films. *Diam. Relat. Mater.* **17**, 1608-1612 (2008).
9. Seo, M. A., et al. Terahertz electromagnetic interference shielding using single-walled carbon nanotube flexible films. *Appl. Phys. Lett.* **93**, 231905 (2008).
10. Polley, D., Barman, A. & Mitra, R. K. EMI shielding and conductivity of carbon nanotube-polymer composites at terahertz frequency. *Opt. Lett.* **39**, 1541-1544 (2014).
11. Macutkevic, J., et al. Multi-walled carbon nanotubes/PMMA composites for THz applications. *Diam. Relat. Mater.* **25**, 13-18 (2012).
12. Das, A., Megaridis, C. M., Liu, L., Wang, T. & Biswas, A. Design and synthesis of superhydrophobic carbon nanofiber composite coatings for terahertz frequency shielding and attenuation. *Appl. Phys. Lett.* **98**, 174101 (2011).
13. Zdrojek, M., et al. Graphene-based plastic absorber for total sub-terahertz radiation shielding. *Nanoscale* **10**, 13426-13431 (2018).
14. Das, A., et al. Quasi-optical terahertz polarizers enabled by inkjet printing of carbon nanocomposites. *Appl. Phys. Lett.* **101**, 910 (2012).
15. Agnandji, E. N., Vigneras, V., Miane, J. L. & Mounaix, P. Shielding effectiveness in terahertz domain of monolayer-doped polyaniline films. *Electron. Lett.* **43**, 1271-1273 (2007).
16. Seo, M. A., Lee, J. W. & Kim, D. S. Dielectric constant engineering with polymethylmethacrylate-graphite metastate composites in the terahertz region. *J. Appl. Phys.* **99**, 066103 (2006).
17. Venkatachalam, S., Bertin, D., Ducournau, G., Lampin, J. F. & Hourlier, D. Kapton-derived carbon as efficient terahertz absorbers. *Carbon* **100**, 158-164 (2016).
18. Lin, Z., et al. Highly stable 3D $Ti_3C_2T_x$ MXene-based foam architectures toward high-performance terahertz radiation shielding. *ACS Nano* **14**, 2109-2117 (2020).

19. Chen, H., et al. Consecutively strong absorption from gigahertz to terahertz bands of a monolithic three-dimensional Fe_3O_4/graphene material. *ACS Appl. Mater. Interfaces* **11**, 1274-1282 (2019).
20. Huang, Z., et al. Graphene-based composites combining both excellent terahertz shielding and stealth performance. *Adv. Opt. Mater.* **6**, 1801165 (2018).
21. Ma, W., et al. Compressible highly stable 3D porous MXene/GO foam with a tunable high-performance stealth property in the terahertz band. *ACS Appl. Mater. Interfaces* **11**, 25369-25377 (2019).
22. Pavlou, C., et al. Effective EMI shielding behaviour of thin graphene/PMMA nanolaminates in the THz range. *Nat. Commun.* **12**, 4655 (2021).
23. Kumar, P., Šilhavík, M., Parida, M. R., Kužel, P. & Červenka, J. 3D graphene straintronics for broadband terahertz modulation. *Adv. Electron. Mater.* **10**, 2300853 (2024).
24. Ji, J., Wang, Y., Zhao, W. & Wang, G. Laser-induced graphene/TiCN on a polyimide/MXene film as interference shielding materials for terahertz electromagnetic waves. *ACS Appl. Nano Mater.* **6**, 23401-23409 (2023).
25. Arooj, N., et al. Polymeric Fe_3O_4 nanoparticle/carbon nanofiber hybrid nanocomposite coatings for improved terahertz shielding. *ACS Appl. Nano Mater.* **6**, 5264-5273 (2023).
26. Hong, X., et al. High-permittivity solvents increase MXene stability and stacking order enabling ultraefficient terahertz shielding. *Adv. Sci.* **11**, 2305099 (2024).
27. Xie, Q., et al. Lightweight MXene-based hybrid aerogels with ultrabroadband terahertz absorption and anisotropic strain sensitivity. *ACS Appl. Mater. Interfaces* **14**, 57008-57015 (2022).
28. Xu, J., Fang, J., Zuo, P., Wang, Y. & Zhuang, Q. Competitively assembled aramid-MXene janus aerogel film exhibiting concurrently robust shielding and effective anti-reflection performance. *Adv. Funct. Mater.* **34**, 2400732 (2024).
29. Kuang, C., et al. Switchable broadband terahertz absorbers based on conducting polymer-cellulose aerogels. *Adv. Sci.* **11**, 2305898 (2024).
30. Theja, V. C. S., et al. 3D architectural MXene-based composite films for stealth terahertz electromagnetic interference shielding performance. *Adv. Mater. Interfaces* **10**, 2300440 (2023).
31. Arooj, N., et al. Optimizing electromagnetic interference shielding of carbon nanofibers reinforced nylon 6, 6 nanocomposite films in terahertz range. *J. Appl. Polym. Sci.* **140**, e53790 (2023).
32. Li, G., et al. Temperature-induced self-decomposition doping of Fe_3GeTe_2 to achieve ultra-high T_c of 496 K for multispectral compatible strong electromagnetic wave absorption. *Adv. Funct. Mater.* **33**, 2210578 (2023).
33. Zhou, C., et al. Porous graphene produced by carbothermal shock for green electromagnetic interference shielding in both microwave and terahertz bands. *Small Methods* **7**, 2201493 (2023).
34. Xiang, M., et al. Flexible composites by ionic liquid/silver/graphene in towel-gourd sponge fibers: synergistic effect and dual-band electromagnetic interference shielding in X-band and terahertz-band. *J. Appl. Polym. Sci.* **139**, e52511 (2022).
35. Bai, Y., Qin, F. & Lu, Y. Flexible and lightweight Ni/MXene decorated polyurethane sponge composite with sensitive strain sensing performance for ultrahigh terahertz absorption. *Adv. Optical Mater.* **10**, 2101868 (2022).

36. Yang, S., et al. Ultrathin, flexible, and high-strength polypyrrole/$Ti_3C_2T_x$ film for wide-band gigahertz and terahertz electromagnetic interference shielding. *J. Mater. Chem. A* **10**, 23570-23579 (2022).
37. Danish, M., et al. Spray deposited graphene@MXene on a flexible polymer substrate for terahertz shielding. *Mater. Chem. Phys.* **311**, 128573 (2024).
38. Pai, A. R., et al. Ultra-broadband shielding of cellulose nanofiber commingled biocarbon functional constructs: a paradigm shift towards sustainable terahertz absorbers. *Chem. Eng. J.* **467**, 143213 (2023).
39. Cao, Y., et al. Multifunctional graphene/carbon fiber aerogels toward compatible electromagnetic wave absorption and shielding in gigahertz and terahertz bands with optimized radar cross section. *Carbon* **199**, 333-346 (2022).
40. Li, S., Xu, S., Pan, K., Du, J. & Qiu, J. Ultra-thin broadband terahertz absorption and electromagnetic shielding properties of MXene/rGO composite film. *Carbon* **194**, 127-139 (2022).
41. Bai, Y., Qin, F. & Lu, Y. Lightweight Ni/CNT decorated melamine sponge with sensitive strain sensing performance for ultrahigh electromagnetic absorption in both GHz and THz bands. *Chem. Eng. J.* **429**, 132393 (2022).
42. Stephan, O., et al. Doping graphitic and carbon nanotube structures with boron and nitrogen. *Science* **266**, 1683-1685 (1994).
43. Nishino, H., et al. Formation and characterization of hydrogen boride sheets derived from MgB_2 by cation exchange. *J. Am. Chem. Soc.* **139**, 13761-13769 (2017).
44. Zhu, H., Wang, X., Cao, Z., Chen, H. & Deng, S. A universal approach for maximizing terahertz wave absorption in graphene cut-wires. *Front. Mater.* **8**, 737347 (2021).
45. Kravets, V. G., et al. Singular phase nano-optics in plasmonic metamaterials for label-free single-molecule detection. *Nat. Mater.* **12**, 304-309 (2013).
46. Dereshgi, S. A., Liu, Z. & Aydin, K. Anisotropic localized surface plasmons in borophene. *Opt. Express* **28**, 16725-16739 (2020).
47. Huang, Y., Shirodkar, S. N. & Yakobson, B. I. Two-dimensional boron polymorphs for visible range plasmonics: a first-principles exploration. *J. Am. Chem. Soc.* **139**, 17181-17185 (2017).
48. Maier, S. A. *Plasmonics: fundamentals and applications*. Springer New York, NY, 2007.
49. Hangyo, M., Nagashima, T. & Nashima, S. Spectroscopy by pulsed terahertz radiation. *Meas. Sci. Technol.* **13**, 1727 (2002).
50. Liu, M., et al. Multi-controlled broadband terahertz absorber engineered with VO_2-integrated borophene metamaterials. *Opt. Mater. Express* **11**, 2627 (2021).
51. Yang, Y., et al. Biomimetic porous MXene-based hydrogel for high-performance and multifunctional electromagnetic interference shielding. *ACS Mater. Lett.* **4**, 2352-2361 (2022).
52. Tripathi, S. N., Saini, P., Gupta, D. & Choudhary, V. Electrical and mechanical properties of PMMA/reduced graphene oxide nanocomposites prepared via in situ polymerization. *J. Mater. Sci.* **48**, 6223-6232 (2013).
